# “It has to be fixed”: a qualitative inquiry into perceived ADHD behaviour among affected individuals and parents in Western Australia

**DOI:** 10.1186/s12913-016-1399-1

**Published:** 2016-04-22

**Authors:** Manonita Ghosh, Colleen Fisher, David B. Preen, C. D’Arcy J. Holman

**Affiliations:** Centre for Health Services Research, School of Population Health, The University of Western Australia, 35 Stirling Highway, Crawley, WA 6009 Australia; School of Population Health, The University of Western Australia, 35 Stirling Highway, Crawley, WA 6009 Australia

**Keywords:** ADHD, Stimulant medication, Qualitative study, Social constructivism, Thematic analysis

## Abstract

**Background:**

The use of stimulant medication for Attention Deficit Hyperactivity Disorder (ADHD) to improve classroom behaviour and sustained concentration is well known. Achieving a better academic grade has been reported as the prime motivation for stimulant use and is an increasingly discussed topic. The proliferation of stimulant use for ADHD has been a cause for public, medical and policy concern in Australia. This paper explores individuals’ perceptions of ADHD, the meaning that the diagnosis carries for them and their attitudes to stimulant medication treatment.

**Methods:**

This qualitative study was underpinned by a social constructivist approach and involved semi-structured interviews with eight participants. The participants were parents of children with ADHD or were adults who themselves had been diagnosed with ADHD. Interviews were audiotaped, transcribed verbatim and thematically analysed.

**Results:**

There were three interrelated yet contradictory overarching themes: (i) An impairment to achieving success, which can be a double-edged sword, but has to be fixed; (ii) Diagnosis as a relief that alleviates fault and acknowledges familial inheritance; (iii) Responsibility to be normal and to fit in with societal expectations. Collectively, these perceptions and meanings were powerful drivers of stimulant use.

**Conclusions:**

Paying attention to perceptions of ADHD and reasons for seeking or not seeking stimulant treatment is important when planning appropriate interventions for this condition.

**Electronic supplementary material:**

The online version of this article (doi:10.1186/s12913-016-1399-1) contains supplementary material, which is available to authorized users.

## Background

Attention Deficit Hyperactivity Disorder (ADHD) is defined as a chronic neuro-developmental disorder characterised by the core symptoms of hyperactivity, impulsivity and inattention [1]. It affects more than 7 % of children [2] with a higher prevalence among boys [3]. There has, however, been considerable argument surrounding the aetiology of ADHD, questioning whether it is a neuro-genetic condition or a socially constructed illness [4]. The debate is fuelled in part by the general if not ambiguous nature of ADHD symptoms defined in the DSM-V [1]. Some argue that children often display behavioural characteristics that include features analogous to the symptoms of ADHD, such as hyperactivity and inattentiveness [5]. Additionally, the treatment of ADHD with psychostimulant medication has been controversial and the focus of much debate in developed countries including Australia, due to the subjectivity of the diagnosis as well as the ethics of treating children long-term with substances that have the potential for abuse [6, 7]. Much attention has also been paid to the short and long-term effects of stimulant use including the risk of cardiovascular complications [8].

Prescriptions for simulant medication have risen sharply in Western countries over the last decade [9–11]. In Australia, the rate of stimulant treatment rose 72 % between 2000 and 2011 [12], representing an average annual growth of 4.7 % and exceeding estimated growth rates in the US by 1–2 % [13]. The persistent rise reflects widening diagnostic criteria [14], an increase in studies purporting benefits from early recognition of ADHD and the efficacy of stimulant medications [15], effective marketing by pharmaceutical companies and a greater acceptance of stimulant treatment among health professionals [16]. This proliferation of stimulant treatment has been cause for public concern that too many children are diagnosed with ADHD and treated with stimulant medications when they do not actually have a disorder [17]. The stimulant medications that are recommended by American Academy of Paediatrics [18] may lessen the severity of ADHD symptoms, increase attention and concentration, and improve classroom behaviour [19]. However, it has been noted that short-term stimulant treatment showing improvement in childhood does not necessarily have lasting effects into the adolescent years [20]. Further, the medications do not promote learning or improve cognitive ability [21]. Nevertheless, it has been reported that they are used for the purpose of neurocognitive enhancement [6]. Parents are more likely to adhere to stimulant treatment if their child has a cognitive impairment [22]. Achieving better academic grades and enhancing cognitive performance is reported to have been the prime motivation for stimulant use among asymptomatic students, who feigned the features of ADHD in order to obtain prescriptions [23].

In light of polarised debate, yet increasing use of stimulant treatment, individuals’ perceptions and experiences related to their child’s or their own diagnosis and treatment are important, but have largely been ignored. Taylor et al. [24] suggested that parents’ decisions to administer psychostimulant treatment are based on their own blend of personal experience, observations of societal norms and media reports. Understanding individuals’ perceptions of and attitudes towards stimulant treatment is important for appropriate intervention and proper management. Understanding which members of the community are more willing to accept stimulant treatment and the factors that make stimulant use more acceptable would be helpful to avoid over-diagnosis and overtreatment.

This study used a qualitative approach to understand how parents of children diagnosed with ADHD, as well as adults diagnosed with ADHD perceived ADHD behaviour and stimulant treatment. The objectives of this study were to explore individuals’ perceptions of ADHD behaviour, the meaning that the diagnosis carries for them, and their attitudes to stimulant treatment derived from their everyday experiences.

## Methods

The qualitative approach used for this research was underpinned by a social constructivist philosophical stance, which emphasized the way individuals seek to understand their world and construct their own particular meanings that correspond to their experiences [25]. It assumes that knowledge and truths are created by the individual’s everyday interactions [26]. Constructivism therefore assisted in the understanding of how the participants in this study perceived ADHD behaviour and stimulant treatment.

### Sample

Participants were recruited through volunteer sampling. An information sheet was distributed to primary schools, Department of Health Western Australia (WA) facilities, and the Learning and Attentional Disorder Society Support Groups in WA. A snowball technique in which participants were contacted via networks was also employed [27]. Participants were selected based on the following criteria [28]: those who themselves were diagnosed or had dependent children diagnosed with ADHD; aged over 18 years; and were able to converse fluently in English. We received expressions of interest to participate in this study from ten people with similar ethnic and socioeconomic backgrounds. Data saturation was reached after collecting information from eight participants as no further new themes emerged from the analysis [29]. The participants were aged 30–60 years, seven of whom were female and all were English speaking and resided in the Perth metropolitan area (see Table 1). The sample comprised six parents of children with ADHD (aged 3–23 years), one grandmother of an ADHD child aged 17 years and one childless adult diagnosed with ADHD and depression. Four parents had more than one child diagnosed with ADHD. Three parents were also diagnosed with ADHD at ages 40–45 years and another two were diagnosed with depression. All participants described their race as Caucasian with five born in Australia and three born in the US, UK and South Africa respectively. Of those born overseas, two had been living in Australia for 22–26 years and the third immigrated 5 years ago prior to starting a family. They all had university degrees, five were professionals, two were stay-home parents and one was a student.

**Table 1:** Demographic characteristics of the study participants

Total participants (*n* = 8)	
Sex	
Female	7
Male	1
Age group in years	
20–40	2
41–60	6
Race	
Caucasian	8
Country of birth	
Australia	5
South Africa	1
United Kingdom	1
United State	1
Education attainment	
Undergraduate	1
Graduate	4
Post graduate	3
Diagnosed with	
ADHD	4
Depression	3
Had child/ren with ADHD	
One child	3
More than one child	4
Had no children	1
Category of respondent	
Mother	5
Father	1
Grandmother	1
Adult with no children	1

### Data collection procedure

In order to understand the individuals’ perceptions towards ADHD behaviour, after obtaining signed consent to participate, semi-structured face-to-face in-depth interviews were conducted to capture participant beliefs and thoughts in their own words [30]. Seven key questions guided the interview sessions were (See Additional file 1 for demographic and interview questions):

1. How was the decision made to seek for professional help?
2. How did you feel being/your child being diagnosed with ADHD?
3. Who do you think is responsible for this condition (ADHD)? Why
4. How was the decision made to administer medication?
5. How do you feel about administering medication for ADHD?
6. How was your/child’s behaviour and your decision to administer medication received by your family and friends?
7. How did their response make you feel? How did you want them to make you feel?

The semi-structured interview kept the discussion on track within the available time restraints. Each interview lasted approximately one hour. This was long enough to encourage participants to talk freely, but not too long to tire them. To keep all interviews consistently focussed yet uninhibited, each participant was asked the same questions, but with a change in the order of questions as appropriate to maintain the flow of the interview. Additional open ended questions were used as prompts, depending on the nature of the discussion. With each participant’s permission all interviews were audio-recorded. The interviews took place at participants’ homes or at other alternative participant-nominated locations to allow them to retain some control over the interview situation and to render the interview session non-threatening, comfortable and convenient for them [31].

### Data analysis

Thematic analysis of participants’ transcribed interviews was conducted. Thematic analysis is a qualitative “method for identifying, analysing and reporting patterns (themes) within data” [32] (P. 79). Thematic analysis is appropriate for exploring and understanding individuals’ experiences, which are often multi-dimensional and multi-layered, whilst elucidating various aspects of the research topic. Braun and Clarke [32] have defined six phases of conducting thematic analysis: familiarising with the data; generating initial codes; searching for themes; reviewing themes; defining and naming the themes; and report producing. Audio recordings of all interviews were transcribed verbatim and the transcriptions were carefully read several times to become familiar with the data to obtain a holistic appreciation of participants’ experiences. Salient words, phrases and sentences used by the participants were highlighted in this phase. Participants’ statements or moments of experience were then initially coded so as to capture their ideas. Next codes were collated into potential overarching themes and sub-themes. In the fourth phase, all overarching themes and sub-themes were then re-checked against the coded data extracts, as well as the entire original transcriptions and refined to ensure an authentic reflection of the participants’ experiences. This phase necessitated a more focused analytical ordering of themes and subthemes. The next phase involved generating definitions and names for each theme to tell the overall story. The final phase of the data analysis consisted of selection of vivid and compelling extract examples, relating the analysis back to the research question and literature. The first author carried out the data collection and data analysis and other co-authors contributed to study design, formulation of data analysis plan and interpretation of findings.

### Rigour

An in-depth face-to-face interview method was used to explore individuals’ perceptions and beliefs towards ADHD and stimulant medication use in their own words. Therefore, the research method was anchored in the constructivist tradition to construct knowledge, meaning and understanding through human interactions, and so the trustworthiness was ensured in the course of conducting this study [33]. Rigour was also enhanced through familiarity with and continual immersion in the data at every step before and during analysis [32]. The validity of individual overarching themes and sub-themes in relation to the data set was ensured through continual revision and checking of coded data extracts and transcriptions, individually and collectively, to reflect accurately the meanings evident in the data as a whole [32]. Producing the research findings to tell the complex story of the participants’ perceptions and experiences was another way to ensure the validity of the analysis [32].

### Ethics approval

The study adhered to ethical principles according to the National Health and Medical Research Council guidelines for conducting human research [34]. Ethical clearance was obtained from The University of Western Australia Human Research Ethics Committee (RA/4/1/2000). As part of this approval, each participant received a written participant information sheet, advising that participation was voluntary and assuring the person that they could decline to answer any question that they felt uncomfortable with and that they were at liberty to withdraw at any time without consequence. The anonymity of the participants was protected by using pseudonyms.

## Results

**Fig. 1:**
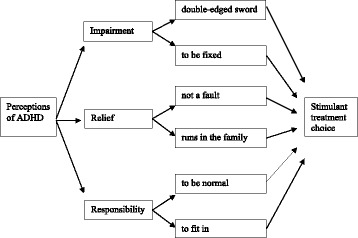
Perceptions of ADHD and stimulant treatment choice. The results consisted of interrelated yet sometimes contradictory themes that depicted the participants’ views of ADHD and stimulant treatment, and in turn influenced the participants’ decision making process to adhere to stimulant treatment

The analysis of the interviews revealed three overarching themes with two sub-themes for each. In sharing their experiences, participants touched on many common themes, yet sometimes these were in indirect contradiction to one another, demonstrating the complexity of the topic. Collectively, the interrelated yet contradictory perceptions and meanings were powerful drivers of stimulant use and are illustrated in Fig. 1.

### An impairment to achieving success

#### It’s a double-edged sword

In discussing their experiences the participants expressed their negative and positive views towards ADHD. Parents reflecting on their experiences of their children suggested that children with ADHD were ‘difficult to manage’, ‘challenging’, ‘exhausting’, ‘not normal’ and had something ‘wrong’ in them. The parents stated that ADHD had a profound impact on their children’s learning at school, and noticed ‘lack of concentration’, ‘struggling with school work’, ‘not achieving at school’ and having ‘no friends’. Some noticed an emotional roller coaster in their children and one felt that her son was suicidal.

*I didn’t realise that the emotional problem that [my son] was experiencing was direct result of ADHD. I knew his hyperactivity was, but I didn’t know the emotional difficulties he had. I didn’t understand the impact of ADHD he had. I just saw the hyperactivity thing … struggling at school … but by the time he was 15, things were coming apart and really I did worry that he would suicide.* (Jo)

As adults, participants described similar experiences including ‘lack of concentration’, ‘unable to remain seated’, difficulties in ‘waking up in the morning’, ‘organising tasks’, and ‘working with co-workers’.

Despite the difficulties they experienced, as parents, the participants often added positive attribution of their child’s ADHD for higher physical energy levels and cognitive abilities. Some believed that people with ADHD could be highly intelligent, and articulated that ADHD symptoms could be seen among famous people like ‘Aristotle’ and ‘Einstein’ throughout history. Annette had two of her children diagnosed with ADHD, and she thought:

*First it was a curse. It was so hard to control; it pulls them so difficult, they torn of their different feelings [sic]. But other side some of the things they do are amazing. It is like a gift, having extra especial power, but whether you can control them?*

Jo perceived a positive side of ADHD along with its negative connotation. She found her ADHD child was fun:

*He was intellectually challenging and fun to rear … interesting … creative so far out of the box … you have to have eyes [in the] back of your head … never a dull moment … exhausting and emotionally scarring but incredibly interesting … intellectual stimulus … you can put [it] that way*.

Jo was also diagnosed with ADHD and taking medications. She compared her life to her friends’ lives, and described:

*They [her friends’ lives] are organized and dull – my one is chaotic and fun. We see more funny connection than other people see – more interesting … one of my friends said I was lot more fun before [treated].*

She then added when she was asked about her perception towards ADHD:

*I think about this a lot and I’m not sure. We read a lot about those outstanding individuals for whom ADHD has been a blessing that they have made great discoveries and they are enormously successful …. There was a belief that human clan would not succeed without ADHD people in the society … [but] the flip side of me says, the success stories that … you do not hear about people who had their life in hell because of the emotional difficulties that ADHD creates. My son and I talked about this a lot. We both agreed [that]… the technicolour as we call the life we live is not worth, we prefer the black & white that everybody lives. So … it’s a double edged sword ….*

#### It has to be fixed

Predominantly, however, ADHD was viewed as a ‘problem’ and a ‘disability’ that needs to be cured. Participants frequently mentioned the impact of ADHD behaviour on their child’s inability to ‘achieve’ at school and ‘function properly’ in society. They believed academic achievement is the key to success in life, and everybody should strive for success. Participants perceived ADHD as an ‘impairment’ to achieving ‘success’. As Susan stated:

*We need to fix the problem. If he is not achieving at school what he is supposed to achieve and there is a problem, then do whatever it needs to be done to fix it …*.

Kate’s perspective was similar:

*We think ADHD is impairment to success. We think success is everything. You got to be successful in life, academically and financially successful. You need to achieve regular goals, family/wife/kids. So, this is impairment, something to be cured … it has to be fixed.*

### Diagnosis as a relief

#### It’s not my fault

‘Diagnosis as a relief’ was the vernacular that all participants expressed knowing that the condition was a ‘neurobiological disorder’ caused by ‘missing chemicals in the brain’, and so was not their fault. The diagnosis relieved them from anxiety and stress, and provided reassurance that they or their children were not ‘bad’ or ‘naughty’, ‘lazy’ or ‘stupid’. The relief was also closely linked with the sense that there is help available, as Cheryl expressed:

*I was pleased to find out that there was something we could do because my son was really struggling. So it was very good for us to find out what the cause was of his problem … a big relief because he was clearly intelligent but he was clearly struggling*.

Linda described her feelings after she was diagnosed with ADHD:

*I actually felt relief. I was relieved because I realised that I was neither mad nor bad. There was certainly evidence that made other people to feel that I was bad … sometimes I thought am I crazy? People said I am rude, I am this and that, I am mad … but I thought I had a good heart … I am concerned about people’s wellbeing and I wanted to help…now I know why.*

The diagnosis served parents as a mean of validating their parenting skills. They felt relief from a burden of guilt about being bad parents who could not discipline their children. As Jo felt:

*… relaxed, thankful, happy, probably validated. Along the way we tried couple of others because he [son] had learning difficulties despite his intellect, we had him tested various other types of psychologically tested. They said he had learning difficulties associated with eye sight tracking which is very common with ADHD … but he had two other diagnoses that both said that I was an over protective mother that caused those hassles… so I was causing his problem. They said I was the problem, not him … it was all my fault and he had nothing wrong with it. So when he was diagnosed very comprehensively to fulfil more than minimum criteria [for ADHD], or maximum whatever you could have, it was a validation for him and for me. For him it was giving a name and understanding of why he was different.*

All participants had a biomedical understanding of ADHD and recognised it as a medical condition, but, did not perceive this condition as a mental illness. As Fiona (grandmother) described her grandson’s ADHD:

*He is missing a chemical that doesn’t allow his brain to do what he needs to do. His brain is seeking stimulation or lacking the ability … it’s not an illness, but it’s a disability, because his body can’t do certain things.*

Kate acknowledged that according to diagnostic criteria – the Diagnostic and Statistical Manual of Mental Disorder [1], ADHD is a mental illness, but still she was reluctant to believe that her son had a mental illness:

*Well, ADHD is a mental health issue in the DSM 4 and 5. According to that he has, but I don’t think about it to be honest … I don’t see him mentally … but if not treated it can be a mental health issue… [so] we accommodate for his [ADHD], so he won’t have any serious mental health [problem].*

While the participants were reluctant to accept ADHD as a mental illness, they accepted this condition as a disability. It appeared that the disability status of ADHD might serve some purpose for two of the participants. Annette who had two of her children diagnosed with ADHD, found that school staff were supportive and they understood ADHD as a disability, and accommodated her children’s needs with special care as necessary. She, however, became “frustrated” with the social security personnel who did not think of ADHD as a disability and so did not think Annette should be eligible for a caregiver allowance for children with disabilities. However, Linda who did not perceive ADHD as a mental health problem, but believed it as a disability, was able to secure her disability pension for ADHD.

#### It runs in the family

The relief was closely tied with perceiving ADHD as a genetic condition and realising that it ran in other family members. The participants mentioned that after one child was diagnosed, they started noticing similar symptoms in their other children and in other family members. Three of the participants were diagnosed after their children were diagnosed. Patrick was one of them:

*My two older boys were diagnosed first, and listening to the paediatricians I realised that me and my wife had the same symptoms … so it came through our lines, therefore it was genetic and heritage ran from us … so we were treated.*

Annette defended herself saying that she tried her best to be a ‘good mother’ during her pregnancy and afterwards doing ‘everything right’ and confirmed that ADHD was not her fault but a genetic disorder.

*When I was pregnant, I did nothing wrong – no drinking, no drug, no bad food. I did everything by the book. I pumped myself from everything to be the best mother … since they were little I gave them boundaries, they were not allowed to have soft drinks, sugar … so, that was not the case … it [ADHD] was from the family … it’s genetic, it’s through the generation … through the father … while doing this [treatment for the first child] we sort of knew my husband had had it for a very long time. So we got him diagnosed … it’s hereditary … since my husband was diagnosed, my father-in-law with full of ADHD … in his 60s … said to me “I know I’ve got it”.*

### Responsibility

#### To be normal

Responsibility focused on parents’ abilities to solve the problem their child was experiencing at school and how stimulant medications helped the parents to fulfil their responsibilities. When describing the effect of medication, the parents and grandmother in the study placed emphasis on their child’s academic outcomes. The medication helped their child to ‘slow down’ and to engage ‘straight away’, which helped the child to ‘focus’, ‘concentrate better’ and ‘stay on task’ at school. The parents frequently mentioned that their child achieved higher grades after taking medications. Three parents said that the school teachers were also happy with the change in their child’s behaviour. For two parents, medications were believed to improve the ‘quality of life’ of the child. A few participants reported unpleasant effects of the stimulants, such as ‘mood swing’, ‘weight loss’, ‘decreased appetite’, ‘heart going faster’ and ‘trouble with sleeping’. They mentioned that their child was prescribed ‘Catapres’, which was usually prescribed for high blood pressure, to reduce the side-effect of the ADHD stimulants. All parents acknowledged stimulants as the most ‘evidence based’, ‘effective’ treatment. Four recognised the stimulant as an ‘important part’ of the ADHD treatment strategy, but as only ‘a part’, and said that their child also required psychotherapy, counselling, learning and behavioural management.

All parents reported in initial hesitation about using stimulant treatment, and expressed concern that the medication was ‘not good for health’. The grandmother mentioned that her initial resistance to medication was due to ongoing debate in the public sphere about over-diagnosis of and over-medication for ADHD. Two parents exhausted other alternative therapies before they agreed to stimulant medication. As Cheryl noted:

*… it’s not the decision that parents make easily. Any long term medication that you put your child on – this is something you have to think about long and hard. You have to decide that your child is diagnosed with something; you have to be satisfied with … pros and cons. … We tried occupational therapy … all sorts of things and wasted lots of money … but when it was clear that none of these were happening - it was already two years. Actually it was my biggest regret that I didn’t try with the medication at the beginning. He would have learnt a lot more at school in those two years.*

Parents felt it was their responsibility to boost their child’s ‘self-esteem’ to make them feel ‘normal’, so that they could ‘fit in’ society. Annette felt ‘strange’ with the idea of giving medication to manage behaviour initially; however, she convinced herself, thinking that giving stimulants to her children for ADHD was similar to taking blood pressure medication for herself. As such, she realised that she must not feel guilty about giving the ADHD medication, because it was her responsibility as a ‘good mother’ to help her children to improve their self-esteem and to allow them to fit into the community. As she articulated:

*I felt strange … but now it is a normal way of life and I don’t have a problem with it. So I don’t feel guilty about giving [medication] to them [the children]. As a parent, it is my responsibility to give them … it is no difference to my blood pressure tablet. I have to take blood pressure tablet or I would feel ill. … So, it’s [medication] a tool for them to cope. To me, it’s a tool to not feel inadequate, not feel different. I don’t want them to be in trouble. I don’t want them to feel that there was something wrong with them … it’s about self-esteem, confidence … about fitting in … learning, adaptation. … So they have their routine, and they have to take the medication every day to be normal.*

In many instances within the interviews the participants defended others’ perceptions of their ADHD behaviour or that of their children. They took a proactive approach to educate people about the nature of ADHD and the role of medication. They tried to move beyond what other people perceived about themselves and also any feeling of guilt that troubled them about giving medication to their child. They appeared to be justifying their decision to adhering medication treatment. They advocated for ADHD as a disability and the use of medication as an acceptable treatment choice. They believed that by educating others they would normalise ADHD, destigmatise stimulant treatment, and improve the outcomes of their and their child’s ADHD. Through this role, they constructed themselves and their child as normal. Patrick and Cheryl took on the position of educators for managing ADHD and stimulant treatment. Annette raised money for an ADHD charity. She and her husband, who was also diagnosed with ADHD, “*proudly wear t-shirts writing on it – ‘ADHD makes me with a super power’, ‘I’ve forgotten my meds today’ so that [their] kids have a role model*”. Annette also worked hard to educate other parents and children about ADHD so that other children would find her children ‘normal’ and ‘average’. As she described:

*So, I try to make it open as much as possible. I brought the book, and the teachers read with other kids that [her son’s name] has a brain, and he can’t concentrate. So the whole class would know – that’s how the teacher is educating the children. There is nothing wrong with [her son’s name], he has taken medication to calm down. So if the parents think that way, it should come from the children that [her son’s name] is not naughty, not a bad kid and he does not have any problem. I used to bring all books and DVD on ADHD and gave it to teachers to give it to other parents, because I wanted to have others to be educated*.

#### To fit in

Susan also expressed her concern about giving medication to her child, but she justified her decision by saying that as a ‘responsible mum’ it was her duty to make her son fit into school and, therefore, she needed to continue with the medication. To minimise the harm of the medication and to allow her son to ‘learn to be himself’, she gave him a break from medication on school holidays.

*I feel I am doing the right thing, but I wish I didn’t have to … I don’t like it [giving the medication], but I have to do it for his education. It’s a drug and it is not good for your body, … so on school holidays I don’t give it to him, he doesn’t need it for any reason, because he is what he is, so he can learn to be himself without the pill … but he has to go to school and do what he is told to do and to fit in. If he was diabetic, he had to have the medication. So I feel to be a responsible mum, he has to have it. I can’t have him the way he wants to be … that’s not responsible parenting … that’s wrong … giving him wrong expectations – so he has to take the pills … responsibility is you have to do what you have to do. If he is happy with his life later not to take it that would be his adult decision, [but] I will guide him until then.*

Susan, however, did not get her older son, who she thought also had ADHD, diagnosed and treated, because his condition was not affecting his education. She thought because he was ‘getting away with his school work’, she did not need to ‘medicate’ him. Through comparing ADHD medication to that taken for chronic diseases, such as diabetics or blood pressure, the parents emphasised the medication’s role in their child’s future – perceiving it as an investment for their child’s academic career, despite giving medication being an unpleasant feeling for them. Thus, they constructed themselves as a ‘good parent’ which allowed them to resolve any feelings of guilt about giving medication.

For the adults with ADHD, taking medication was also about a responsibility to ‘fit in’ at work, within family and in relationships. They experienced improvement on every day activities, organising tasks at work, interpersonal skills and having meaningful conversations like ‘normal people’. They found their family and friends coped better with them when they were on medication. As such it was their responsibility to fit in with family and society. As Susan, who was also taking medication for ADHD, reflected:

*I take my pill to cope with pressure … I could cope with the world … if I didn’t take the pill I wouldn’t be able to talk to you like this … wouldn’t be able to focus at work … my husband copes better with me when I am on the pill … so I have to [take medication], to stay in the relationship, to keep our life easy.*

## Discussion

The findings of this exploratory study assist in understanding the complexity of ADHD. The results consisted of interrelated yet sometimes contradictory themes that depicted the participants’ views of ADHD and stimulant treatment. There were three overarching themes: an impairment to achieving success, diagnosis as a relief and responsibility. The findings from this study reflect the ontological and epistemological assumptions of the social constructivist framework [35], which assumes that across individuals there may be multiple understandings of phenomena, being in this instance ADHD and attitudes towards its treatment with stimulant medication. It was notable that the themes were defined and redefined by the participants particularly through their everyday interactions with others in a community setting.

ADHD is perceived to be an impairment to achieving success reflected in two sub-themes: ‘it’s a double-edged sword’ and ‘it has to be fixed’. The expression ‘double-edged sword’ in this study bares similarity to observations made in a UK study, where Singh et al. [36] noted that young people with ADHD expressed a dichotomous sense of themselves. They felt that their ADHD behaviour was ‘fun’, but then acknowledged that their fun behaviour was ‘annoying’ to others. The participants in this study perceived ADHD as fun, challenging and interesting; however, when comparing the fun behaviour with the perceived obstacle to achieving success, and particularly academic success, they chose to accept ADHD as an impairment which had to be ‘fixed’.

The participants had a biomedical understanding of ADHD, ascribed to it a causal relationship with academic under performance [37] and accepted stimulant medication as the eventual treatment of choice, albeit sometimes after seeking alternatives or as the mainstay of a broader treatment strategy. This was in line with a help-seeking behaviour model for ADHD, which suggests that the individual’s perceptions about ADHD influence their treatment choice [38]. Our findings resonate with those from Canada, where Johnston et al. [39] identified that people’s degree of acceptance of a medical aetiology of ADHD was significantly associated with stimulant treatment choices. The participants in this study were Caucasians, relatively affluent and well educated. Understanding ADHD as a medical condition and accepting stimulant treatment to improve academic performance is comparatively more common among Caucasian families than in other ethnic groups in the US and UK [6, 40]. However, when a child’s academic achievement seemed to be threatened by ADHD, people from other cultural and ethnic background were also found to accept stimulant treatment. Korean parents who tended to take personal responsibility for their child’s ADHD behaviour and had initial negative attitudes towards medication treatment, were reported to administer stimulants once they believed that ADHD was associated with their child’s relative lack of academic achievement [41]. In a study in India, the findings suggested that parents who resisted a biomedical explanation of their child’s ADHD behaviour tended at first to seek religious help to minimise the impact of the ADHD. The same parents, however, sought medical interventions when they perceived that problems with their child’s academic performance were not improving [42].

Participants in this study described a sense of relief following diagnosis, as it provided them with an explanation for the difficulties they or their child had experienced. The sense of relief stemmed from the fact that the diagnosis reassured them that the problem behaviour was not a personal failing in any moral sense, but rather a mental or at least cognitive disorder. In a phenomenological study of eight adults with ADHD in the UK, Young et al. [43] also reported that the diagnosis eliminated an individual’s sense of failure as their ADHD behaviour could be explained and attributed to a specific disorder. The parents in this study tended to defend themselves, saying that they did their best to be ‘good’ parents and so the parenting was not the cause of their child’s ADHD. As such, the diagnosis validated the child’s problem behaviour and school failure what not a reflection of failed parenting. These findings have parallels in another study by Singh [44], conducted in the UK and US among 153 children and their parents, for whom the diagnosis provided a great relief. Parents (mothers especially) with an ADHD child often walk a fine line between perceiving themselves as ‘good’ and ‘bad’ parents, because they are most often blamed for their child’s misbehaviour and under achievement [45–47]. Hence, scholars argue that although parents use the biomedical model of aetiology of ADHD to provide some relief from parental blame, the medical model may not serve to provide total relief from feelings of personal responsibility, stress, anxiety and guilt [44, 48]. As Taylor et al. [24] noted in an attempt to make the right decision for their child about the stimulant treatment, parents go through several stages in which they face contradictory societal attitudes, such as parental blame for their child’s misbehaviour on one hand, while also experiencing anxiety and guilt feeling about drugging children by administering stimulants. To cope with the stressors of raising ADHD children and the associated societal pressures, parents tend to defend themselves by employing strategies like advocacy, education and strategic difference [49], all of which are congruent with the findings from this study.

Parental concern about the potential long-term side effects of stimulant medication was observed in another study in the US, where parents of children with ADHD expressed fears and accepted stimulant treatment reluctantly, even though they agreed that the medication helped their child [50]. Despite being worried about long-term effects and some immediate unpleasant side-effects of stimulant medication, parents in this study felt that it was their responsibility to continue with the medication to improve their child’s self-esteem by helping the child feel normal and to ‘fit in’ with the community. Whilst results have been reported where parents discontinued stimulant treatment in their child’s best interest due to uncertainty about long-term effects on brain function, related stigma and the child’s disliking of use [51], the present findings are more consistent with those of Hansen and Hansen [48], who found that parents tolerated the medication’s side effects and risks in the hope that it would play an important role in enabling the child to attain their academic goals and achieve success in their adult life.

Parental effort to improve their child’s academic performance and boost self-esteem is a rational consequence of the fact that higher education is a critical path to one’s career success in modern society [52, 53]. To achieve career success, an individual is required to develop competency across a wide range of personal qualities, which may include self-esteem [54, 55]. While some investigators have found no influence of self-esteem on relationship or career success [56, 57], others claim that a high level of self-esteem is crucial for success and life satisfaction in these spheres [58–60]. Whether one’s self-esteem serves career success or not, self-esteem is valued in today’s society [61], and developing children’s self-esteem is reportedly evident as a cornerstone of contemporary Western parenting practices, particularly in middle class families [62]. In a qualitative study using semi-structured interviews with Canadian parents of children seen as cognitively impaired (including some with ADHD and learning disabilities) and those with unimpaired children, Ball and Wolbring [22] found that parents would consider stimulant use if they perceived their child was struggling at school, failing to fit in or had low self-esteem. The parents in their study believed that it was their responsibility as good parents to make their child feel normal and encourage them to succeed in life.

The adults diagnosed with ADHD in this study also referred to their needs to feel ‘normal’, to have the ability to interact with other people, to belong in the community and to be accepted by family and friends. These findings are consistent with other studies where adults with ADHD perceived that being accepted by others as a normal, responsible social being was important [43, 63], and that the medication enabled a sense of normality and social belonging to occur [43, 64].

The findings from this study suggest that individuals’ perceptions and experiences shared much in common in the general sense, yet in detail the individuals’ journey had been diverse and complex. The findings underline that a person’s understanding of ADHD behaviour and their attitude towards stimulant treatment are important considerations in selecting an appropriate intervention and in developing policy on the regulation of stimulant treatment use. Individuals who do not experience the perceptions of difficulties in academic performance or fitting in with society may not necessarily seek stimulant treatment, even if it would be beneficial from an objective viewpoint. This was noted in the interview with Susan, who thought that her older son also had ADHD, but did not seek treatment for him because he was achieving school grades to her satisfaction. On the other hand, desire to accelerate academic performance may motivate individuals to pursue the non-medical use of stimulants [65, 66]. Parents were hesitant to use stimulant medication initially due to long-term side effects, but administered it as they were concerned about their child’s academic under performance, self-esteem or failing to be ‘normal’. More parents may consider stimulant medication if they perceive these drugs as less harmful or if cultural trends redefine what is normal.

This study contributes to the body of literature with its focus on individuals’ perceptions of ADHD and attitudes towards stimulant medication, including perceived roles of medication in child’s future. Paying attention to perceptions of ADHD and reasons for seeking or not seeking stimulant treatment is important when planning appropriate interventions for this condition to avoid over-diagnosis and overtreatment. The findings reinforce the need for more education of medical professionals to enable them to plan appropriate interventions and to give appropriate support and guidance to optimise outcomes for individuals with ADHD and their families.

There are some limitations to this study that deserve consideration. Firstly, the participants were white, middle class people, living in a metropolitan area. As such, their perceptions may not reflect those of community members from other backgrounds, highlighting the need for research among culturally, ethnically and socioeconomically diverse groups in the future. Secondly, as the sample was largely female, the views of a wider range of males were somewhat absent from the research. Thirdly, three of the four adults with ADHD were parents who were diagnosed after their children had been diagnosed, and mostly described their experience as being parents. Even though the experience of one adult with no children was little different from the parents diagnosed with ADHD, findings drawn from this sample may not be transferrable to the perceptions of ADHD and stimulant treatment of adults diagnosed with ADHD. Fourthly, given the nature of qualitative analysis, this study represents only one interpretation of the participants’ experiences, hence it delivers a partial, static picture of their perceptions of ADHD behaviour and attitudes towards stimulant treatment. Further, the analysis primarily denotes interpretations made by a single research group with the possibility that others might draw different inferences. Despite its limitations, this study does provide some important data with respect to the factors that shape individuals’ attitudes towards ADHD and influence individuals’ treatment choices. Building on these insights, further research can be conducted in a format that would canvas a wider range of views. Future research could also include a multi-perspective and longitudinal design, interviewing children, young adults and their parents to explore evolving perceptions of ADHD and medication over time.

## Conclusion

The participants in this study perceived ADHD behaviour as an impairment to achieving success in life. A desire for academic achievement, good self-esteem, being normal and a sense of belongingness were important driving forces for stimulant treatment use among parents of children diagnosed with ADHD. Adults diagnosed with ADHD found stimulant medication was important for a responsible person to fit in to the community. The findings have potential to be used to raise awareness and understanding among medical practitioners working with ADHD adults, children and their parents of the perceived reasons why individuals seek or do not seek stimulant treatment.

### Availability of data and materials

In accordance with the Human Research Ethics Committee, University of Western Australia (HREC-UWA) protocol, research data involving human participants cannot be made available to the public for confidentiality and ethical reasons. Demographic and interview questions are included as an additional file.

## Additional file

Additional file 1: Demographic and Interview Questions. (DOCX 36 kb)

## Abbreviations

ADHD: Attention Deficit Hyperactivity Disorder
WA: Western Australia
