# Supplementary material for: “It has to be fixed”: a qualitative inquiry into perceived ADHD behaviour among affected individuals and parents in Western Australia
Source: BMC Health Serv Res. 2016 Apr 22;16:141. doi: 10.1186/s12913-016-1399-1 (PMC4840935; doi:10.1186/s12913-016-1399-1)
Supplement: Additional file 1: — Demographic and Interview Questions. (DOCX 36 kb) [file 12913_2016_1399_MOESM1_ESM.docx]

# Demographic Questionnaire

1. Date questionnaire completed________________

2. Your age _____________________

3. Age of your child/ren _____________________

4. Number of children _____________________

5. Your occupation _________________________

6. Name of your suburb ___________________________

7. Marital status: (circle your answers)

Single married never married remarried

divorced widowed de-facto

8. Are you involved in any community work? e.g. sports, cultural program Y/N

9. If yes, what do you do? ______________

10. Is your child involved in any activities? e.g. sports, cultural program Y/N

11. If yes, what does she/he do? ______________

12. What is your partner’s occupation? ______________

13. What is your country of origin? ____________

14. What is your ethnic identity? ____________

15. What languages do you speak? ____________

16. How long have you been in Australia? ____________

17. Are you an Australia citizen? Y/N

18. If no, what is your resident status in Australia?

Permanent resident temporary resident visitor other ________

19. What visa category did you have when you came to Australia?

Permanent resident spouse family reunion

Refugee other ________

20. Did you want to come to Australia? Y/N

21. Do you want to stay in Australia? Y/N

22. How many of your family live with you in your house? _________

23. How many of your family live nearby? __________________

24. Where do they live? (tick your answers)

Next door next street same neighbourhood next suburb other__________________

25. Do you have any relatives or friends who help you? Y/N

26. What kind of help do they give?

Financial emotional house work other _____________

27. Have you ever been diagnosed with ADHD? Y/N

28. If yes, at what age were you diagnosed with ADHD? _____________

29. Have any of your children been diagnosed with ADHD? Y/N

30. If yes, how many of your children have been diagnosed with ADHD? __________

31. At what age were your children diagnosed with ADHD? __________________

32. Do you have any other family member diagnosed with ADHD? Y/N/Not sure

33. If yes, how many of your family member is diagnosed with ADHD? _________

34. If yes, what is the relationship with you? __________________

35. At what age were they diagnosed with ADHD? __________________

36. Did you take any prescribed medication for ADHD? Y/N

37. Did your child take any prescribed medication for ADHD? Y/N

38. At what age did you take your first medication? __________________

39. At what age did your child take first medication? __________________

40. What medication were you prescribed? __________________

41. What medication was your child prescribed? __________________

42. How long have you been taking medication? ____________

43. How long has your child been taking medication? ____________

44. Do you/your child use any alternative treatment for ADHD? Y/N

45. If yes, what treatments do you/your child use? __________________

**Thank you for your participation**

**Interview Questions**

1. When did you first notice this condition? What did you think about it?
2. What was your experience, physically and emotionally when this issue began?
3. How was your/child’s behaviour received by your family and friends?
4. How do you compare your life to your friend’s life?
5. What made you seek professional help?
6. How was the decision made to see a psychiatrist or paediatrician?
7. What was the procedure to see a psychiatrist or paediatrician?
8. How often do you need to see the specialist?
9. How do you/your child feel to visit the specialist?
10. How did you feel being/your child being diagnosed with ADHD?
11. What do you think about ADHD?
12. How do you describe ADHD as an illness or a mental health issue?
13. Who do you think is responsible for ADHD? Why? How?
14. How do you feel being a parent?
15. How do you deal with the reality?
16. How was the decision made to administer medication?
17. How did/do you feel being/your child being prescribed a medication for ADHD?
18. What medication was prescribed?
19. What was the procedure in terms of taking medication?
20. What is your experience about the medication doses?
21. Did you want to take the medication? Why/why not?
22. How do you feel about taking medication for ADHD or giving to your child?
23. How do you think the medication may help you/your child for ADHD?
24. What is your experience after taking medication?
25. What did you think about possible side-effect of the medication?
26. What is your experience of the costs associated with the treatment?
27. How was your decision to administer medication received by your family and friends?
28. Where do you get most advice/support from?
29. How do you perceive what other people think about you?
30. How do you cope with your feelings?
31. How do you want them to see you?
32. Do you think your experience would be same if you were in your home country?
33. How do you think people in your home country would see ADHD?
34. How do you describe your ethnic background?
35. Do you think your ethnic background may influence the way you think about ADHD?
36. Do you think your socio-economic background may influence the way you think about ADHD?
37. Do you have any other comments you want to add?

**Thank you for your participation**
